# Designing and implementing equity-based pandemic preparedness and response learning modules: lessons from a multi-country short-course

**DOI:** 10.1080/16549716.2022.2104319

**Published:** 2022-08-12

**Authors:** Anatole Manzi, Phaedra Henley, Hannah Lieberman, Langley Topper, Bernice Wuethrich, Jenae Logan, Abebe Bekele, Joel Mubiligi, Sheila Davis, Agnes Binagwaho, Paul Farmer, Joia Mukherjee

**Affiliations:** a Directorate of Clinical Services, Partners In Health, Boston, MA, USA, Kigali, Rwanda; b Division of Global Health Equity, Brigham and Women’s Hospital, Boston, MA, USA; c Department of Global Health and Social Medicine, Harvard Medical School, Boston, MA, USA; d One Health Division, University of Global Health Equity, Butaro, Rwanda; e Department of Quality and Patient Safety, Tufts Medical Center, Boston, MA, USA

**Keywords:** Pandemic response, pandemic preparedness, equity, online course, COVID-19

## Abstract

**Background:**

The COVID-19 pandemic has had disproportionate impacts across race, social class, and geography. Insufficient attention has been paid to addressing the massive inequities worsened by COVID-19. In July 2020, Partners In Health (PIH) and the University of Global Health Equity (UGHE) delivered a four-module short course, ‘An Equity Approach to Pandemic Preparedness and Response: Emerging Insights from COVID-19 Global Response Leaders.’

**Objective:**

We describe the design and use of a case-based, short-course education model to transfer knowledge and skills in equity approaches to pandemic preparedness and response.

**Methods:**

This course used case studies of Massachusetts and Navajo Nation in the US, and Rwanda to highlight examples of equity-centered pandemic response. Course participants completed a post-session assessment survey after each of the four modules. A mixed-method analysis was conducted to elucidate knowledge acquisition on key topics and assess participants’ experience and satisfaction with the course.

**Results:**

Forty-four percent of participants identified, ‘Immediate need for skills and information to address COVID-19’ as their primary reason for attending the course. Participants reported that they are very likely (4.75 out of 5) to use the information, tools, or skills from the course in their work. The average score for content-related questions answered correctly was 82–88% for each session. Participants (~70-90%) said their understanding was Excellent or Very Good for each session. Participants expressed a deepened understanding of the importance of prioritizing vulnerable communities and built global solidarity.

**Conclusion:**

The training contributed to a new level of understanding of the social determinants of health and equity issues surrounding pandemic preparedness and response. This course elucidated the intersection of racism and wealth inequality; the role of the social determinants of health in pandemic preparedness and response; and the impacts of neocolonialism on pandemic response in low- and middle-income countries.

## Background

The beginning of 2020 was marked by the declaration of the COVID-19 pandemic as a Public Health Emergency of International Concern. As the virus spread, the world feared the health and economic impacts but paid little attention to addressing the massive inequities worsened by COVID-19. Amidst the challenges of COVID-19 response, there were a few places, including Massachusetts and Navajo Nation in the US and Rwanda that prioritized equity in their response strategy by emphasizing connecting affected people with social support and protection. In the US, COVID-19 has had disproportionate impacts across race, class, gender, and geography and has laid bare deep disparities within the US health system [1,2]. Black, Indigenous, Latinx, and other people of color are most affected by COVID-19 and carry a greater burden of cases, hospitalizations, and deaths relative to their share of the population. In the US, the mortality rate of Black people due to COVID-19 is double the rate of white people [3]. Low-income and minority communities are more likely to be exposed to COVID-19 as they are more likely to hold essential service sector jobs, to live in high-density housing, and to depend on public transportation [4]. These same groups have higher rates of pre-existing underlying health conditions, less access to healthcare services, more limited insurance coverage, and less financial freedom to stay home to quarantine [5,6]. Incomplete data prevents us from understanding the full extent of the disproportionate impact of COVID-19 [3].

For those who study and practice social medicine, it is not surprising that the pandemic epicenter emerged first in the US, where the factor most significantly associated with a shortened life span is zip code or residence address. Zip code encodes centuries of slavery, stolen native lands, forcibly outlined reservations, decades of Jim Crow, red-lining, impoverished school systems, failed public housing projects, unsafe working conditions, and mass incarceration [7]. The difference in life expectancy between the poorest communities in the US and the richest ones is about 30 years [8]. This is similar to the differences in life expectancy between the US overall and a low-income country like Liberia [9].

In China, a country with near universal health coverage, there were early warnings of inequities in the impact of COVID-19 [10]. It was immediately recognized that the elderly were far more likely to die from COVID-19 and that those with pre-existing conditions, such as chronic heart disease, diabetes, and chronic respiratory illness, had higher fatality rates [11]. What was less publicized was that people with COVID-19 coming from poorer and polluted districts in China and Italy had poorer outcomes [12–14]. In South Africa, COVID-19 highlighted persisting racial inequities [15]. Urban areas in South Africa remain racially divided and mainly Black townships have experienced high rates of COVID-19 [16]. In South Africa, as in India, Peru, and the US, it is challenging for community members living or working in overcrowded, underserviced areas to adhere to physical distancing recommendations.

Differences in risk, severity, and outcome of diseases are always linked to the social determinants of health [17]. Epidemiologists and clinicians will fail to control epidemic diseases if health equity is not at the root of the response. Infectious diseases, crowded living conditions, lack of water and sanitation, food insecurity, and lack of access to timely health care are major drivers of epidemics [17].

The quality of national leadership becomes crucial during a pandemic. Leadership that is science-based and compassionate and focused on protecting the most vulnerable through equity-centered action is crucial for a successful pandemic response [18]. Countries that have adhered to these principles have more successfully curbed outbreaks and lowered death rates, while those that have belittled science and privileged the better-off in society with treatment and prevention have seen pandemics spiral.

Equity is a basic building block of pandemic preparedness, namely well-funded systems of prevention and care that are consistently available and affordable to all. Health practitioners must not just do clinical work; they must act intentionally to counteract inequity. Additionally, public health and social service stakeholders must recognize their co-responsibility. In fighting COVID-19, the most vulnerable people cannot follow guidelines for stay-at-home, quarantine, and isolation unless they receive social and material support [8]. COVID-19 exposed the need for additional competencies among public health implementers, health professionals, and policymakers, highlighting equity as a core determinant of effective pandemic preparedness and response (PPR).

Several organizations including John Hopkins and World Health Organization designed and implemented webinar presentations or short courses that focused primarily on epidemiological surveillance, contact tracing, and clinical case management, without explicit attention to the equity dimension of PPR [19,20]. To address this critical need, Partners In Health (PIH) and the University of Global Health Equity (UGHE) organized a four-session virtual course, ‘An Equity-Approach to Pandemic Preparedness and Response: Emerging Insights from COVID-19 Global Response Leaders,’ targeted at leaders and implementers responding to COVID-19 globally.

## Intervention description and design

A case-based instructional approach was adopted to deliver a coherent analysis of equity-based PPR and practical competencies in equity-based PPR. Targeted participants included public health implementers, clinicians, and policy makers from around the world. To ensure equitable participation and representation, social media posts were circulated at least a month prior to the course. The invitation and social media posts were in English and Spanish. To account for time differences, all sessions occurred at 9:00am EST. All participants were informed of the content and evaluation structure as part of the introductory session. All participants who completed their post-session survey were included in this evaluation. The course aimed to evaluate essential practices to address pandemics and epidemics, to assess and improve tools for COVID-19 response, to analyze strategies to integrate health equity, and to discuss lessons learned in effective design and implementation of COVID-19 interventions. In addition to a global overview of the dimensions of past and present pandemics, the course team developed three case studies highlighting COVID-19 response in Massachusetts and Navajo Nation in the US, and Rwanda. The content emphasized centering community leadership in agenda-setting and intentionally driving resources towards the most vulnerable, acknowledging that communities are made vulnerable due to the forces of structural violence, racism, and neoliberal capitalism.

Using interactive case studies, the PIH and UGHE short course focused on equity approaches to PPR. The course involved the faculty, expert speakers, and participants with extensive experience and global perspectives. This was crucial to disseminate adaptable and scalable practices. The course consisted of four sessions, each with a 1.5-hour presentation and 1-hour live Question & Answer session. These presentations provided further clarifications of case studies and other pre-reading materials. To accommodate geographic time differences and schedules, participants were given an option to view the recorded sessions off-line. Participants who watched all four sessions and completed the four post-session assessment surveys received a certificate co-signed by the organizing institutions.

Course development was guided by four core approaches that were highlighted in each case: 1) prioritize strategies to address basic needs, 2) focus on vulnerable communities, 3) understand healthcare as a human right, and 4) strengthen leadership and accountability. Table 1 describes core approaches which guided the development and delivery of the course content.

**Table 1.** Core approaches for the development and delivery of the pandemic preparedness and response course.

Approach	Description
Meet Basic Needs	It is impossible to adhere to prevention strategies if you have no food, money, or housing.
Prioritize the Vulnerable	Target resources at vulnerable communities.Examples: assure food security & housing, provide transportation & cash
Engage Those Most Affected	Community members who are most affected and have a central role to play in responding to crises. Example: Contact tracing helps to stop the spread of epidemics and is best done by community members. When this work is adequately compensated, it fulfills a double purpose of reaching the most vulnerable and adding jobs in a difficult financial environment.
Healthcare as a Right	Support for the public provision of healthcare prior to, during, and after an epidemic is the most durable form of pandemic preparedness.
Accountable Leadership	Leadership should be accountable for the outcomes of the most vulnerable and listen to communities to understand what is needed.Leaders need to build trust over time with their communities.

## Course structure

This short course was offered in four modules over a period of two weeks in July 2020. Case studies were informed by a series of interviews with experts in global health, public health, social medicine, history, and equity in the US, the Navajo Nation, Rwanda, Liberia, Sierra Leone, Lesotho, Peru and Haiti. They were drafted and edited by lead writers, and collectively and individually reviewed and critiqued by key experts, and the PIH/UGHE implementation team. A hybrid approach was adopted to deliver the course content, which consisted of a combination of readings, webinars, and didactics. The 1.5-hour sessions were held on Zoom and were each followed by a 1-hour Question & Answer (Q&A). Each session began with a panel discussion featuring five to seven speakers who were pandemic experts, public health leaders, and frontline implementers. Each module focused on case studies, illustrating core principles of a health-equity approach to PPR. Table 2 describes the content and specific learning objectives. Each session concluded with an ‘Office Hour,’ which provided participants with the opportunity to submit questions to the panelists through Zoom’s chat function. Participants were encouraged to attend the live sessions but had the option to view the recording after. Prior to each session, pre-readings about the case studies and equity frameworks were disseminated to participants to provide background knowledge and allow participants to focus on the speakers.

**Table 2.** Course content overview.

Session	Learning objectives
Session 1: The 21st Century Pandemic: Covid-19 And Health Equity7 July 2020	This session provided an overview of the equity-based lens and how it can be used to guide COVID-19 response. Review the origin, evolution, and early warning indicators of COVID-19 and global inequality. Understand the legacy of inequality within and among countries past pandemics Discuss leadership dimensions for effective preparedness and response to Covid-19 and other pandemics Understand the relationship of clinical care, social support and public health in pandemic preparation and response Understand basic principles for developing and sustaining an equitable and effective pandemic response.
Session 2: Contact Tracing and Equity: the Role Of Wrap-Around Support in MassachusettsJuly 9, 2020	This session described the process of establishing a contract tracing program in Massachusetts, USA. With the onset of COVID-19, the Massachusetts Department of Public Health partnered with PIH to develop a robust contact tracing program that has a strong emphasis on CRC. The MA CTC program is a model for other programs in the U.S.A. Review the pillars of effective contact tracing and understand the importance of wrap-around supports Understand the role of public health leaders in planning pandemic response founded in equity Discuss preliminary lessons, successes, and challenges of Covid-19 contact tracing Understand the types of social support needed for safe and effective isolation and quarantine Describe clinical care referral pathways for contact tracing and their role in promoting equity
Session 3: Covid-19 & Health Equity: Perspectives of Frontline Implementers in the US and the Navajo NationJuly 14, 2020	The Navajo Nation was among the communities hardest hit by COVID-19. In the Navajo Nation, PIH has partnered with tribal leadership and other partners to prioritize resources and address pressing community needs, such as elder support and supportive isolation for multigenerational housing. Review the current state of equity and its implications on public health response to Covid-19 in the US. Discuss a potential theory of change to strengthen health equity integration in the US and globally Understand the impacts of history and COVID-19 on the Navajo Nation, and the ways in which the Nation has organized itself to protect its people and combat the pandemic Describe and discuss the role of public health implementers, policy makers, and civil society Understand the roles and responsibilities of current and future leaders in eliminating social disparities in the context of pandemic preparedness and response
Session 4: Equity & Innovation: The Response to Covid-19 in RwandaJuly 16, 2020	The session was led by response leaders in Rwanda and described Rwanda’s successful response to Ebola and now COVID-19. Illustrate the critical importance of interdisciplinary collaboration and coordination to address problems in global health delivery Discuss how Rwanda used community education to contain COVID-19.. Critically evaluate the challenges facing low-resource settings during the pandemic and after it through an equity lens Discuss the significance of data-driven decision-making and response. Investigate the strategic investments needed to continue care Recognize the importance of social mobilization to support the marginalized. Evaluate the Government of Rwanda’s response to emerging infectious diseases

The course was offered for free and at a time that was convenient for time zones across the Americas and throughout Africa. The workload of the course was designed to be appropriate for individuals that were participating in this course simultaneously with full-time commitments as students or professionals. After each webinar, participants completed a post-session survey that collected basic demographic data, evaluated comprehension of course learnings on key topics, and assessed participants’ experience and satisfaction. At the end of the course participants who had attended all four modules (live or recorded) and completed the assessment surveys received a certificate.

We describe the use of a case-based, short course education model to widely disseminate and transfer knowledge and skills in equity approaches to pandemic preparedness and response for medical professionals, public health implementers, leaders, and policy makers. We review the post-course assessment surveys completed by participants in order to understand what participants gained from this course, how learnings will be applied to participants’ work or studies, and reflect on lessons learned for future PIH and UGHE courses and PPR courses more broadly. Evaluation of the design and implementation of the PIH and UGHE short course offers a potential model for other organizations seeking to offer equity-focused PPR education modules.

## Methods

We conducted a descriptive mixed-method study using the responses of the post-session survey distributed to all participants after each of the four sessions. The surveys were hosted on Microsoft Forms. All responses were gathered July 7–27 July 2020. The surveys ranged in length from 31 to 33 questions, depending on the session. Surveys included: seven demographic questions, six Likert scale questions assessing quality of course components, 3–5 open-ended questions about the impact of the course and suggestions for improvements, a set of five content questions to assess knowledge comprehension, and nine questions for participants to self-assess their individual knowledge acquisition.

## Data collection

**Figure 1.**
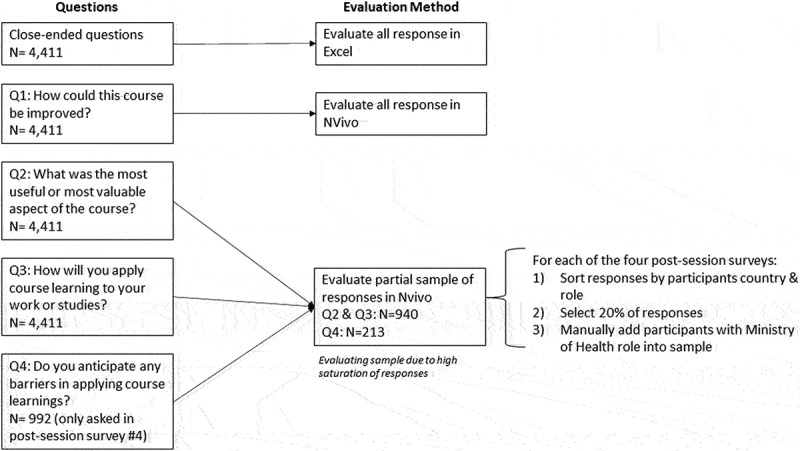
Evaluation and sampling frame.

The four surveys differed only in the content questions about session-specific material. The final survey asked two additional open-ended questions about the application of course content to participants’ professional work and studies (Figure 1). The total number of survey respondents was 1,208, 1,202, 1,009 and 992 for the first, second, third, and fourth session, respectively.

## Data analysis

Descriptive statistics including frequency and percentages were used to understand participants’ demographics, attendance, and knowledge acquisition. Survey data in Excel spreadsheets were imported to NVivo 12 Plus for data analysis of open-ended responses and a list of thematic codes was developed. The initial coding structure was not exhaustive and additional codes were added iteratively throughout several revisions of the coding. Open-ended questions required manual coding in NVivo. For Question #1 (‘How could this course be improved?’), 100% of responses or 1,208 data items were coded. For Question #2-4 (‘What aspects of this course are most useful?’, ‘How will you apply course learnings?’, ‘What do you anticipate the greatest challenges will be to apply or implement the course learnings in your work or studies?’) a sample of 22% of responses (N = 940, N = 940, N = 213) was coded.

## Results

**Figure 2.**
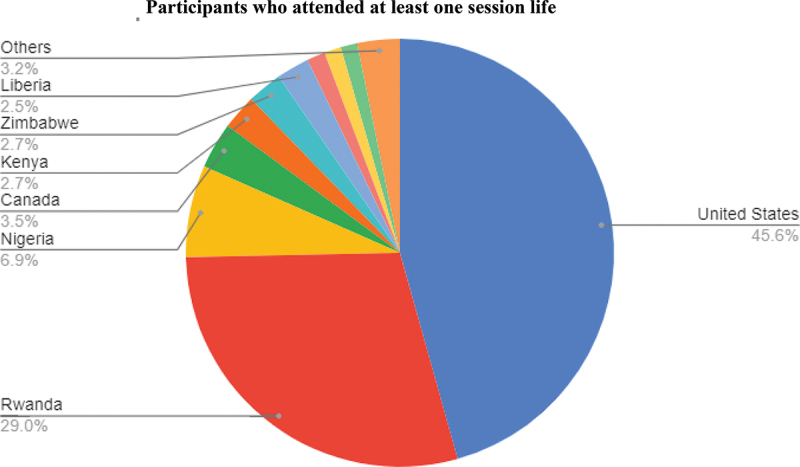
Participant country.

In total, 2,532 individuals registered for the course. On average, a live session was attended by 993 people and 1,608 participants watched at least one live session. In the end, 987 participants received certificates (Table 3). Participants represented 72 countries. Many participants were from the US (47.8%), where PIH is headquartered, and Rwanda (21.2%), where UGHE is located. Approximately 40% of participants were from different African countries (Figure 2). A range of professionals and students attended the sessions, 17% were public health implementers, 21% were medical professionals, 11% were public health leaders, and 27% were students (Table 4). Survey data identified the motivation for attending the course, participants’ prior exposure to equity and racial dynamics, and areas of course success.

**Table 3.** Participant session attendance.

Session	Live Viewers (zoom report tracking viewers)	Viewed Recording (self-reported in post session survey)	Successfully Completed Post Session Survey	Course completion rate
Session 1: The 21stCentury Pandemic:Covid-19 And HealthEquity	1,292	324	1,449	90%
Session 2: Contact Tracing and Equity: MA	1,002	332	1,116	84%
Session 3: COVID-19, Inequity and Racism in the U.S and How the Navajo National is fighting COVID-19	862	371	1,102	89%
Session 4: Equity and Innovation: The Response to COVID-19 in Rwanda	818	366	1,062	90%

**Table 4.** Demographic characteristics of course participants (N = 1062).

Position	Number of participants	% of participants
Students		
Undergraduate	112	10.5%
Masters	80	7.5%
Medical School	70	6.6%
Doctoral	15	1.4%
Secondary School	4	0.4%
Other students	250	23.5%
Medical Professional		
Physician	98	9.2%
Nurse	30	2.8%
Paramedic professional	19	1.8%
Clinical Officer	6	0.6%
Physician’s Assistant	4	0.4%
Other medical professionals	64	6.0%
Implementers	183	17.2%
Public health leaders	113	10.6%
Policy makers	15	1.4%

Source: Self-reported on 4^th^ session

## Motivation for attending

Immediate need for skills and information to address COVID-19 and explicit focus on an equity approach were the primary drivers of participant’s interest in this course, 44% and 27%, respectively.

## Educational background on race & equity

When asked whether their undergraduate, graduate or health professional classes explicitly addressed issues of racism and equity, 29–35% agreed (lower for health professional training), 20–34% disagreed (lower for graduate studies), and 31–45% said not applicable (N/A) (highest for health professional studies). The largest numbers of participants were from the US and Rwanda. A higher percentage of participants from the US both agreed (40% vs. 34% of Rwanda participants) and disagreed (34% vs. 23% of Rwanda participants) that their undergraduate studies explicitly addressed issues of racism and equity. A higher portion of respondents from Rwanda said N/A (42% vs. 27% of US participants). When asked whether their academic studies covered the social determinants of health, but stopped short of naming racism, a higher proportion (46%) agreed, 28% disagreed, and 25% said N/A.

## Areas of course success

Three areas including high comprehension, use of acquired knowledge, alignment with the implementation, and smooth logistics were highlighted as the major areas of success.

*High Content Comprehension*: Overall, participants demonstrated a good understanding of course material. The average score for content-related questions answered correctly was 82% for Session 1, 85% for Session 2, 88% for Session 3, and 86% for Session 4. Participants reported a good grasp on the learning objectives of each session. For Session 1 ~ 70% said their understanding was Excellent or Very Good, ~80% for Sessions 2 and 3, and ~90% for Session 4.

*High Desire to Apply Knowledge*: Participants reported that they are very likely (4.75 out of 5) to use the information, tools, or skills from the course in their work.

*Implementation Team Alignment*: There was strong alignment between the PIH and UGHE contributors, resulting in cohesive course themes, PPR resource sheets, web page resources, and social media content.

*Course Logistics*: When reflecting on the structure and logistics, the course implementation team found that positive aspects were Zoom as the webinar platform, the length of each session, the pre-readings, the dynamic exchange among panelists, and the opportunity for an extended discussion during Office Hours that directly followed panel discussions.

Qualitative findings highlighted practical application of the course, focusing on equity and racism, communication and community engagement, and supportive environment as emerging themes.

## Practical application of the course

For many participants the most useful aspects of this course were the case studies and practical applications. Participants noted either the case studies generally or a specific case study as the most useful aspect of the course.

A medical professional from the US shared, ‘I liked hearing from leaders in Rwanda, where you can see the success that comes from focusing on vulnerable populations.’ When asked how they would apply course learnings to their work or studies, many respondents mentioned they would use learnings to implement best practices. However, some respondents noted that a lack of funding or resources was a barrier to implementing the approaches discussed in the course. Participants were interested in including more case studies generally, with an added focus on low-middle-income country contexts. Many participants also wanted more practical applications. One medical professional from the US shared, ‘I would like to hear more about specific ways healthcare providers can create change in vulnerable communities’ and a public health leader from Zimbabwe said, ‘[I would like to learn more about] practical and specific references to what low-income countries need to do within the available resources.’

Several participants noted that the course challenged them not only to reconsider approaches to PPR in their own contexts, but also to question their prior assumptions and cognitive framework. One participant shared: ‘I found myself thinking of ways to apply the mechanism of countries who invest more into their public health structure to my own community policies that specifically disproportionately affect those who are socioeconomically disadvantaged.’ Another participant said, ‘I loved hearing [course faculty] Dr. Agnes’ perspective and other global leaders in health equity as I have a narrowed U.S. perspective that operates in the systemic inequities built in the U.S.’. Similarly, a participant shared, ‘I also love understanding the pandemic from a different perspective; understanding that not all parts of the world have the level of healthcare inequity that the US has.’ Another participant noted, ‘The cost of poor health system contributed more to my learning and awakened my mind to these problems.’

For many, the discussion of clinical and containment nihilism (views that efforts to either treat or contain the pandemic were pointless), strongly resonated. One participant said: ‘I have been witnessing “containment nihilism” for weeks, and this gave me a name for it, and a reminder to push back on that framework and fight for better.’ Said another, ‘It gave me a great perspective and helped me to articulate my own experiences in a better way.’ Equally appreciated was the clear definition of leadership as being accountable to the most vulnerable in society.

At a time when some of the world’s most prominent leaders were promoting disinformation, belittling science, disregarding the pandemic’s impact on the most vulnerable, and even contributing to increased violence against people of color through their rhetoric, the course and the panel discussions were able to present a fundamentally different vision of leadership and equity in PPR. Many participants expressed the relief and encouragement they experienced through their participation in the course.

## Focus on equity and racism

Participants left this course with a deeper understanding of the importance of approaching PPR with an equity focus. When asked about the most useful or valuable aspects of the course, the equity focus was frequently mentioned. A public health implementer from the US shared, ‘I have a better understanding of the disparities around the world and also have acquired some language to talk about the impact on vulnerable communities, about what an equitable response might look like, and about why and how to invest in social, economic, and health systems.’ A medical professional from Rwanda stated that, ‘[this course reinforced for me] the role and responsibilities of current and future leaders to eliminate social disparities in the context of pandemic preparedness response.’

When asked how they would apply course learnings to their work or studies, respondents mentioned they would use learnings to work toward social equity and many respondents commented that this course was a catalyst to continue personal reflection and education about health equity and pandemic response. A public health implementer from Malawi said this course reminded them of the need to ‘consider inherent inequalities when designing community responses to minimize propagating the inequality.’ Some respondents commented that a barrier to implementing equity-based approaches is their communities’ lack of understanding about the need for an equity focus or willingness to take such approaches. A medical student from Germany shared, ‘a lack of understanding for the need to address social determinants will certainly pose a hindrance in implementing such measures in my future work setting,’ but emphasized ‘I intend to always take a step back and consider the social determinants to make sure I am making equitable decisions and programming.’

One of the four course segments focused on the intersection of racism and the pandemic in the US When asked about the most valuable aspects of this session, a public health leader in the US wrote, ‘Having such an open discussion about equity and racism within the sphere of public health with such passionate public health professionals was empowering.’ A Masters student of governance in Germany replied, ‘The ability … to explicitly address racism denial in health equity and understanding frameworks of racism on institutionalized, personally mediated, and internalized levels helped understanding … bias and privilege.’ Hundreds of people expressed similar sentiments. Many commented on gaining a deeper understanding of racism, including ‘as a system of structuring opportunities based on social interpretation,’ of a person’s value, and of racism as a public health crisis. Some participants from outside the US expressed surprise and concern. A public health worker in Zimbabwe wrote ‘Learning that racial inequalities still exist in developed countries like the U.S. was an eye opener as an implementer in Africa I always felt this is present only here and is fueled by corruption and mismanagement of public resources that widen disparities and leave the poor poorer and the rich better off.’ Hundreds more wrote of how valuable they found the focus on the Navajo Nation’s experience of the pandemic. One US public health implementer wrote: ‘Including the case study on the Navajo Nation was truly brilliant!! No matter how much I hate to admit it: how the reservation was fairing in the battle against Covid-19 never entered my mind.’

## Communication and community

Participants benefited from the opportunity to hear the global perspectives of the panel and ask panelists questions directly. The panel discussion and Q&A were mentioned by many participants as the most valuable aspect of the course. Additionally, participants mentioned the building of community solidarity and opportunity to hear global perspectives. A participant from Senegal shared, ‘I loved how frank the discussion and presentations were. The speakers spoke from lived experiences and with passion about equity, not just as an academic topic, but something they actually believe in and advocate for.’ A student from the US shared, ‘I think it was extremely useful that we got to hear from people in different areas of the public health sector and how they’ve dealt with health equity wherever they may be globally.’ Another student expressed, ‘Connecting with people [from] different countries who talk of their experience in this pandemic was great if one wants to adjust and explore different things that [one] can do to respond.’ A student from the UK shared how meaningful it was to, ‘Finally be able to hear another perspective outside of the U.K., where I live, or the U.S.’

Students also noted the tenor of the conversation. Said one, ‘Being able to ask questions to the panelists and also hearing them ask questions to each other – it is amazing to see leaders also learning from their peers.’ One student noted that the most valuable part of the course for her was, ‘Listening to the speakers discuss and qualify their positions with each other in a constructive and educational way, unlike what we unfortunately are hearing every minute of every day in the news.’

Participants suggested future courses include more panelists who are frontline workers or beneficiaries of COVID-19 programs. In general, participants wanted even more time and opportunities to interact and ask panelists questions directly. Respondents gave feedback on the Q&A format, suggesting allowing participants to pre-submit questions, showing the questions being asked, and distributing a document after the session with responses to unanswered questions.

Many respondents indicated that they would share course learnings with others, and some emphasized they would try to increase collaboration in their work or studies. A public health implementer from Liberia stated that they plan to, ‘share acquired knowledge through practical demonstrations and also encourage and motivate others to emulate this.’ Some respondents expressed that obstacles to sharing course concepts included feeling unsure how to incorporate learnings into their work or discussions with colleagues. When asked about the greatest challenges to applying course learnings to their work or studies, respondents mentioned resistance from political leaders or the political system and institutional barriers of their organizations or society in general. An implementer from Mexico shared that ‘[a challenge is the] governance and support from our government. It has been very challenging to collaborate, especially [with] the Ministry of Health.’ A public health leader from Nigeria anticipated the greatest challenge would be, ‘breaking institutional frameworks that aid this inequity.’

## Supporting participant learning

*Pre-Reading*: For some participants the pre-readings were an important aspect to introduce the topic and offer additional case study examples. One participant from Canada shared that ‘the pre-reading materials were great informational guides and provided well-needed context behind COVID-19.’ Twenty respondents suggested more reading, as compared to only five respondents who recommended less pre-reading. Participants advised further linking the panel discussion to the pre-reading. A public health leader from the US proposed, ‘tying more of the readings into the slides or panel discussion.’

*Visual Aids*: Slides and graphics were identified by participants as a useful tool for communicating information. A medical professional from Mexico suggested ‘having more visual aids’ and a participant from the US said, ‘I wish we had the slides in advance so we can take notes on/alongside them.’ Respondents also indicated that their understanding would have been enhanced by a session summary, one participant commented, ‘maybe have a slide or two at the end with the key takeaways from the discussion.’

*Technical & Logistical Aspects*: Many respondents mentioned technical challenges such as the lighting, framing, volume, sound quality of panelists, and video editing. Participants commented that accessibility could be improved by including the speaker’s titles in their name banner, enabling closed captions for the live session and non-English subtitles for the recorded version, defining technical terminology, slowing the speed of conversation, and providing pre-readings as downloadable pdfs.

## Discussion

The PIH and UGHE short course was distinct from other webinar and course offerings in its equity-focus, case-study-based format, and global presenters and participants. The PIH and UGHE short course focused entirely on equity-based approaches in every aspect of pandemic response and emphasized bringing high-quality care to limited resource communities, whereas other courses discussed equity as one of many considerations in pandemic preparedness or contact tracing.

The delivery of the PIH and UGHE short course on 7–16 July 2020 occurred four months after the declaration of COVID-19 as a global pandemic. This was a timely dissemination of best practices in PPR. Recommendations were evidence-based on newly available medical studies and informed by the course implementation’s deep understanding of historical context and persistent gaps in access, treatment, public health education, and social determinants.

Adopting a case-study approach was a major strength of the PIH and UGHE short course as it provided participants with clear, implementable examples of equitable approaches to PPR. Participants re-framed and reaffirmed their understanding of equity-based approaches and the importance of prioritizing vulnerable communities as central to an effective pandemic response strategy. For many participants, issues of race and equity were not included in their undergraduate, graduate, and/or health professional training, and thus short courses provide a valuable means for continuing education.

An additional strength of this course is that PIH and UGHE approached COVID-19 as a global issue and affirmed the need for global sharing of resources and success stories. Many other PPR webinars and trainings had US-centric content, whereas the PIH and UGHE short course had a global focus, panelists with experience working in many contexts, and a significant global audience made up of various types of providers, decision makers, and community leaders. With its global focus, panelists, and audience, this short course presented equity-focused interventions in a broad range of contexts.

This course showed that high-income countries can learn from the successes of low-income settings, such as Navajo Nation and Rwanda, that have better contained and responded to COVID-19. Further, participants had the opportunity to ask questions during the Q&A to the panel of public health leaders and medical professionals who have upheld and demonstrated the importance of equitable approaches to healthcare during their careers.

The main outcome of the course was that participants had an increased understanding of what equity-based approaches to pandemic preparedness and response look like in practice. A secondary outcome reported by participants was a feeling of community solidarity. Participants felt supported being surrounded by the panelists and fellow participants who are each working to lead their communities and countries through the COVID-19 pandemic.

We recognize three major areas for improvement for future online short courses. The first is making the course more interactive and giving participants more time to ask panelists questions directly. The second is involving a broader range of panelists who are frontline responders and members of affected communities. Third is improving technical aspects, such as lighting, framing, volume, sound quality of panelists, and video editing, which can interfere with participant learning.

A review of other evaluations of short courses shows that the methodology used to evaluate this PIH and UGHE short course was in line with standard practice in measurement and that similar outcomes were achieved [21–23].

This study has some limitations. First, we used data from participants who joined live sessions or self-reported watching the recorded version of the session. As with any online course, the ability to verify attendance was limited. However, it is unlikely that participants would have passed the final exam without having a clear attention to the course content. Additionally, the geographic and time differences meant that some participants watched the course live and had the opportunity to ask questions while others watched a recorded video afterwards. We did not assess whether knowledge acquisition, retention, or course impact differed between the participants who watched live and offline. However, offering an offline viewing option allowed a wider and more global audience to learn about equity-based approaches to PPR. Another limitation is that this survey was distributed immediately following the course. Participants noted how they intended to apply course learnings and what they anticipated would be challenges to apply course learnings.

While online learning approaches have limited effect on knowledge acquisition compared to face-to-face or blended courses [24], participants of this course expressed a remarkable increase in knowledge and skills acquisition. A convergent mixed methods study is needed to assess the level of application and perceived impact of this course from the perspective participants and communities, direct recipients of PPR-related interventions led by participants. This course capitalized on interactions covering practical tools and tactics to ensure equity-centered preparedness and response to pandemics. We believe that real-time interactions with the speakers and collective interest in this timely topic have contributed to increased content comprehension and application. However, rigorous observational studies are needed to measure whether participants of this course demonstrate better decisions and stronger integration of equity principles in their routine practices.

Overall, the course feedback was overwhelmingly positive. The content of the course was applicable to a wide range of individuals in different roles in different countries. A case-based, short course format complimented by live video webinars is an effective way to improve medical professionals, implementers, and public health leaders’ understanding of equity-approaches to PPR. Participants’ interest in the course content demonstrated that high-income countries could learn from the successes of low-income settings, such as Navajo Nation and Rwanda, that have better contained and responded to COVID-19.

## Conclusion

The course was offered in July 2020, as the US solidified its position as the epicenter of the COVID-19 pandemic, and as the Black Lives Matter movement transformed the national discussion of racism, prompting deeper consideration of structural racism and meaningful change, and triggered global waves of support for Black Lives Matter in the wake of George Floyd’s murder. The PPR course addressed and drove awareness of the intersection of these world-shaping events, making the short course, ‘An Equity Approach to Pandemic Preparedness and Response’ an opportunity for participants to draw new connections in real time, while also deriving applicable strategies and tactics for strengthening equity-centered to PPR.

Case-based, practical-application short-courses that incorporate interactive live discussion from cross-cultural and geographically diverse expert presenters, with ample opportunity for engaged dialogue with the audience, are a valuable method for future trainings that can shape fundamental understanding around emerging issues and inspire the adoption of new practices. By speaking to the moment of crisis in real time and bringing together a range of global, theoretical, and practical experience, a course can open minds to new perspectives and modes of action. Participants particularly appreciated the opportunity to hear expert speakers from different parts of the world not only present based on their lived and learned experience, but also interact with each other in dynamic and respectful discourse.

This course used case studies from Massachusetts, Navajo Nation, and Rwanda to offer participants practical examples of centering a PPR around equity. It incorporated a multitude of examples from African countries through both the pre-readings and the sharing of panelists’ experience. Case-based learning in combination with live webinars illustrated what equity approaches to pandemic response can look like in practice and built participants’ competencies alongside content knowledge. Such case-based, hybrid short-courses are a valuable method for future training around emerging pandemics.

## Paper context

To respond to COVID-19, several organizations implemented webinar series that focused primarily on epidemiological surveillance and clinical case management, without explicit attention to equity dimension of the response. A four-session virtual course, ‘An Equity-Approach to Pandemic Preparedness and Response: Emerging Insights from COVID-19 Global Response Leaders,’ targeted leaders and implementers responding to COVID-19 globally and strengthened the pandemic preparedness and response. Findings emphasize that equity as an essential building block of pandemic response.
